# Supplementary material for: Conditional chemoconnectomics (cCCTomics) as a strategy for efficient and conditional targeting of chemical transmission
Source: eLife. 2024 Apr 30;12:RP91927. doi: 10.7554/eLife.91927 (PMC11060718; doi:10.7554/eLife.91927)
Supplement: Figure 3—source data 3. [file elife-91927-fig3-data3.zip › Figure 3 source data3 part 1.zip!/Figure 3-Source data 3-part1/1175-9-CRISPResso_Report_rtnd62ko.zip!/CRISPResso_on_1175_10/1c.Alignment_barplot.pdf]

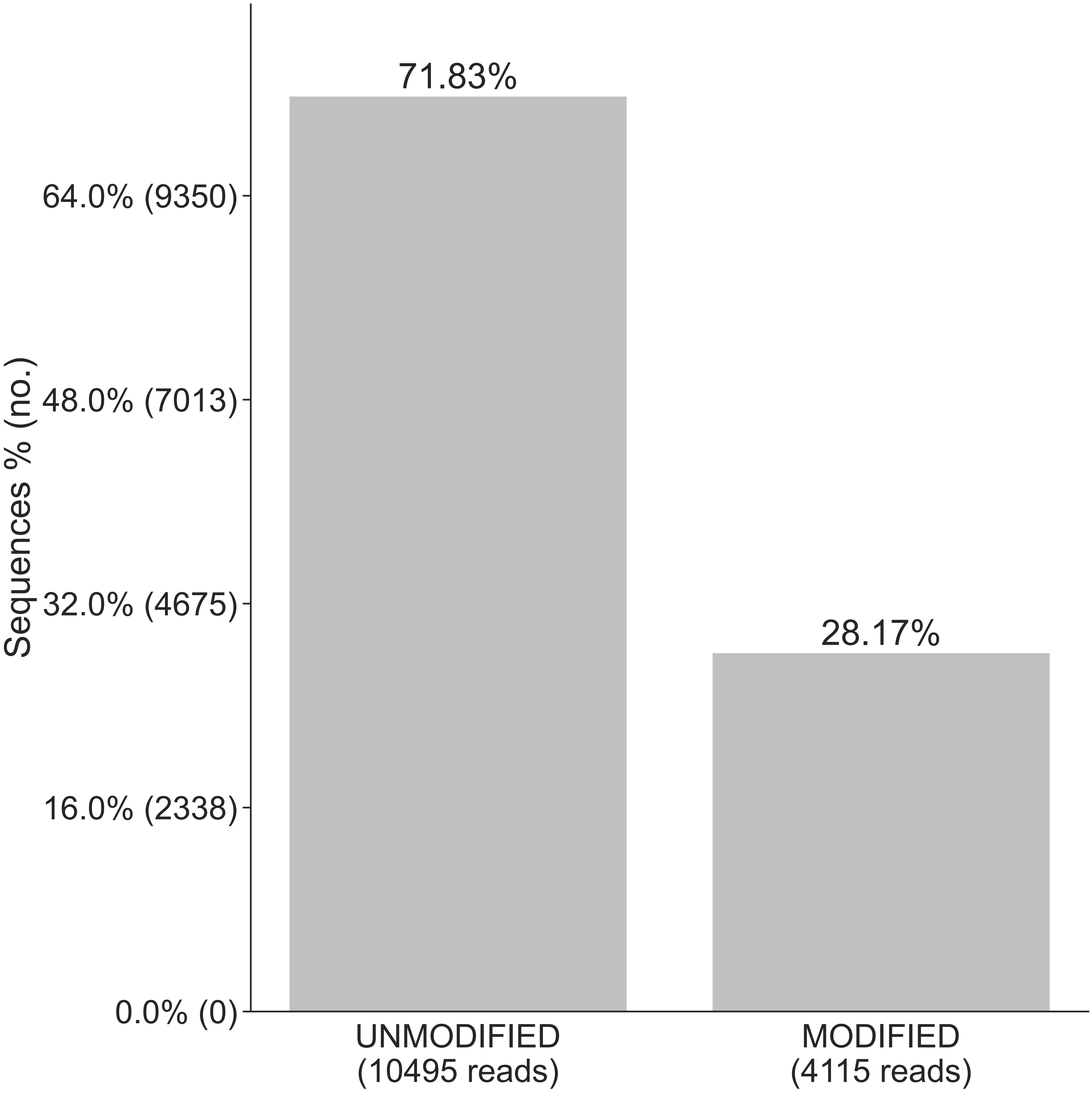

Sequences % (no.)
64.0% (9350)
48.0% (7013)
32.0% (4675)
16.0% (2338)
0.0% (0)
71.83%
28.17%
UNMODIFIED
(10495 reads)
MODIFIED
(4115 reads)
